# Chemokines and Heart Disease: A Network Connecting Cardiovascular Biology to Immune and Autonomic Nervous Systems

**DOI:** 10.1155/2016/5902947

**Published:** 2016-05-03

**Authors:** Veronica Dusi, Alice Ghidoni, Alice Ravera, Gaetano M. De Ferrari, Laura Calvillo

**Affiliations:** ^1^Department of Cardiology and Cardiovascular Clinical Research Center, Fondazione IRCCS Policlinico San Matteo, 27100 Pavia, Italy; ^2^Center for Cardiac Arrhythmias of Genetic Origin, IRCCS Istituto Auxologico Italiano, 20095 Milan, Italy; ^3^Department of Molecular Medicine, University of Pavia, 27100 Pavia, Italy; ^4^Institute of Cardiology, Department of Medical and Surgical Specialties, Radiological Sciences, and Public Health, University and Civil Hospital of Brescia, 25123 Brescia, Italy

## Abstract

Among the chemokines discovered to date, nineteen are presently considered to be relevant in heart disease and are involved in all stages of cardiovascular response to injury. Chemokines are interesting as biomarkers to predict risk of cardiovascular events in apparently healthy people and as possible therapeutic targets. Moreover, they could have a role as mediators of crosstalk between immune and cardiovascular system, since they seem to act as a “working-network” in deep linkage with the autonomic nervous system. In this paper we will describe the single chemokines more involved in heart diseases; then we will present a comprehensive perspective of them as a complex network connecting the cardiovascular system to both the immune and the autonomic nervous systems. Finally, some recent evidences indicating chemokines as a possible new tool to predict cardiovascular risk will be described.

## 1. Introduction

Chemokines are a subgroup of cytokines with the specific function of chemoattraction (*chemo*tactic cyto*kines*) and a molecular weight between 7 and 12 kDa. They are small molecules of approximately 70 amino acid residues derived from a single ancestral gene about 650 million years ago [1]. Chemokines are characterized by several features including the presence of four conserved cysteine residues and “vertical” receptor signal transduction. These receptors often promote rapid and reversible changes in cellular metabolism or migration, in contrast to “horizontal” cytokines receptors which usually cause slower and irreversible cellular changes, such as proliferation and apoptosis [2, 3]. Members of the chemokine family are divided into four groups depending on the spacing of their first two cysteine residues: CC chemokines, which have two adjacent cysteines, CXC chemokines, with the two cysteines separated by one amino acid, C chemokines, characterized by the presence of only two cysteines, and CX3C chemokines with three amino acids separating the initial pair of cysteines.

Among the fifty-two chemokines [4] discovered to date, nineteen are presently considered to be relevant in heart disease, seven of them belonging to the class of CC chemokines, ten to the CXC family, one to the CX3C chemokines, and one to C chemokines.

**Figure 1:**
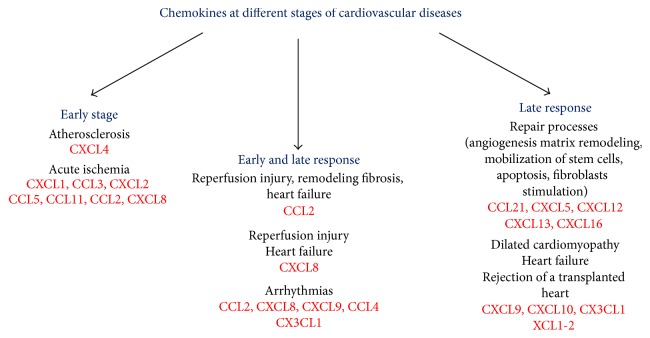
Association between chemokines and pathological conditions present in cardiovascular diseases. Some chemokines play a role in the early stages of cardiovascular disease, being associated with atherosclerosis and acute myocardial ischemia. CCL2 and CXCL8 are involved in both early and late response to ischemic injury, recruiting leukocytes after acute ischemia and playing a role in heart failure. Moreover, together with other chemokines, they are strongly associated with cardiac arrhythmias, a dangerous event which may occur at all stages of the disease. Finally, there are chemokines frequently found in the late response to injury and in the repair process and chemokines more associated with dilated cardiomyopathy and chronic rejection of a transplanted heart.

Some chemokines play a role in the early stages of cardiovascular disease like atherosclerosis or acute ischemia [5–9]. Others are involved in both early and late response to injury and are strongly associated with cardiac arrhythmias, heart failure, and chronic rejection of a transplanted heart [10–22] (Figure 1). The chemokines associated with cardiovascular disease discussed in the present review are listed in Table 1.

## 2. Cardiovascular Disease, from Ischemia to Heart Failure

Myocardial ischemia (MI) is a state of myocardial impairment due to an imbalance between the level of coronary perfusion and myocardial energy demand. Although atherosclerosis is a central part of cardiovascular disease, reviewing the role of chemokines in its development is beyond the scope of the review. We will discuss chemokines involvement in cardiovascular disease from the first event which changes dramatically the metabolism and the homeostasis of the heart, that is, myocardial ischemia.

**Figure 2:**
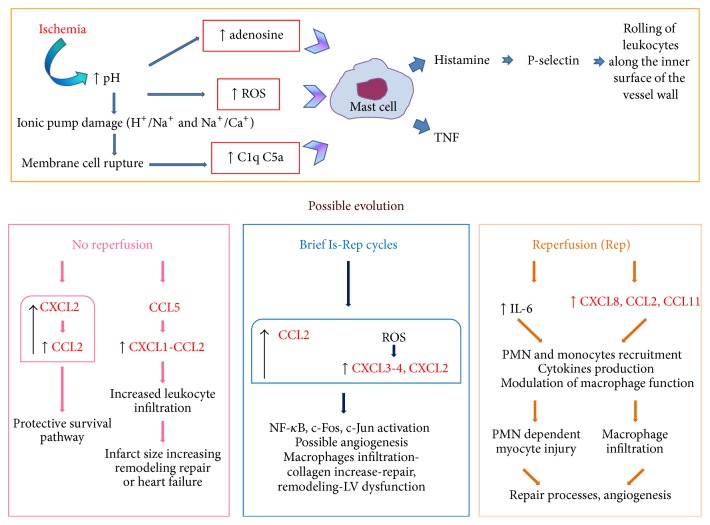
Metabolic changes triggered by ischemic insult and possible evolutions of the injury with the crosstalk between chemokines. The pH decrease, provoked by ischemia, is the event turning on the process. The cell membrane is damaged and debris activates the classic complement pathway in the infarcted myocardium. ROS, adenosine, and complement activate mast cells to produce TNF and histamine, leading to leukocyte recruitment from the vessels. Depending on the presence or the absence of reperfusion, there is a different crosstalk between chemokines, aimed at restoring the balance. Dysregulated or exaggerated responses may actually lead to a progression of the disease (see text for details, chemokines in red).

The mechanical work of the myocardium is driven by aerobic metabolism, which produces energy in the form of adenosine triphosphate (ATP). Myocardial ischemia is followed by the progression from reversible to irreversible myocardial cell injury within about thirty minutes. After this period, histologic features of coagulation necrosis appear, with nuclear pyknosis, karyolysis, and exudative inflammation. Sudden death of the whole heart as such does not occur; every cell of the heart singularly responds to the injury (ischemia) and to the removal of the injury (reperfusion) with biochemical changes moving toward life or death (Figure 2). The severity of the damage depends on time, on the coronary artery involved, and on the entity of the inflammatory response. Two different scenarios are possible after ischemia: permanent occlusion or reperfusion. In case of permanent occlusion, potential causes of irreversibility are catabolism without new synthesis of macromolecules, reduced transmembrane gradients of Na^+^ and K^+^, Ca^2+^ overload, catabolite and oxygen radicals accumulation, enzyme denaturation, cell swelling, and membrane rupture [23]. Early reperfusion, on the other hand, is associated with rapid cellular infiltration of neutrophils and monocytes mainly into the jeopardized border zone surrounding the infarcted area [24]. Neutrophils migrate from the perivascular area and remain primarily in the border zone whereas monocyte migration into the infarct proceeds very rapidly [25–27]. A possible evolution of both scenarios is cardiac hypertrophy, a thickening of the ventricular walls in the heart, which is an adaptive response to pressure overload, volume stress, mutations of proteins involved in the structure or loss of cardiomyocytes, and contractility, all possible consequences of myocardial ischemia. Hypertrophy can be physiological, present as normal response to healthy exercise, characterized by a concentric aspect, or pathological, leading to remodeling and changes in the shape and structure of the myocardium. Concentric hypertrophy is believed to have a compensatory function by diminishing wall stress and oxygen consumption [28] whereas pathological hypertrophy can lead to heart failure (HF) and is associated with major arrhythmias. Pathological hypertrophy activates the expression of genes related to collagen, chemokines, and actin which are not stimulated by physiological hypertrophy [29]. Myocardial hypertrophy is associated with persistent inflammation, irrespective of the cause of the disease. Indeed a similar pattern of inflammatory activation is present in patients with aortic valve stenosis or MI, as well as in animals subjected to different stimuli leading to the development of pathological hypertrophy [19, 30–32]. At the cellular level, cardiomyocyte hypertrophy is characterized by an increase in cell size, enhanced protein synthesis, and heightened organization of the sarcomere [33]. This pathological condition is associated with fibrosis development, characterized by monocytes infiltration and activation of matrix-metalloproteinase (MMP) in extracellular matrix (ECM). In animal models, profibrotic genetic pathways are activated early, before hypertrophic remodeling [36]. Myocardial fibrosis is a hallmark of hypertrophic cardiomyopathy and can evolve in decompensated remodeling, with myocytes slippage and wall thinning [37, 38]. If the loss of viable myocardium has been massive, wall stress increases further and the final result can be left ventricular (LV) dilation and HF a condition in which cardiac function is seriously impaired. This vicious “circle” results in increased blood pressure and LV mechanical overload, which further worsens the condition.

Chemokines seem to act as a “working-network” instead of a “one chemokine-one function” way, in modulating this cardiac response to injury. This characteristic is well known in the field of immunology and is one of the causes of the typical redundancy seen in several inflammatory processes. The complexity of this network is far from being completely understood and described, but recent studies are trying to reassemble the pieces of the puzzle. Recently [39] a small clinical study for risk stratification (a tool for identifying and predicting which patients are at high risk of cardiovascular events) performed in patients after MI evaluated the prognostic significance and the clinical relevance of a cluster of 27 serum cytokines, including the chemokines CXCL8, CCL11, CCL2, CCL3, CCL4, and CCL5. The authors did not identify a single inflammatory cytokine capable of predicting adverse events in a long term follow-up, but they suggested that the presence of more than 13 cytokines above the median value was useful for risk stratification. Another clinical study [40] enrolled 28 patients undergoing PCI, 20 undergoing coronary angiography, and 28 healthy controls, with the aim of analyzing the plasma levels of CCL2 and CCL11 in all groups. The results showed a significant increase of both CCL2 and CCL11 in patients suffering heart disease as compared to healthy subjects. Moreover, the plasma levels of the two chemokines rose significantly after PCI; notably CCL2 rose after 3 and 6 months following PCI and CCL11 rose 24 h but not 3 months after PCI. When the process degenerates toward failure, the inflammatory pathway appears to be almost the same irrespectively of the initial cause of pathology.

Some of the most common signaling pathways [41], relevant in the crosstalk between innate immune response and ischemic heart, include the following:

- TLR-4, responsible for macrophage homing and inflammatory cytokine production;
- HMGB1/TLR-4 for monocytes recruitment and activation of CXCL12-CXCR4 pathway in the leukocytes;
- gp130/JNK/STAT3 and TLR-4/myD88/CAMKII/NF-*κ*B for monocytes and monocyte/neutrophils recruitment and activation;
- cGMP/eNOS, through CXCL12-CXCR4 axis, for neutrophils recruitment and matrix remodeling after ischemic injury.

These pathways underline the presence of communication between cardiovascular and immune systems through several mediators including chemokines. Therefore, we will discuss in detail their role in the progression from ischemia to heart failure.

### 2.1. CCL2, CCL3, CCL4, and CXCL2

CCL2 (Monocyte Chemoattractant Protein or MCP-1) has been the subject of numerous investigations in the field of cardiovascular research. It is produced by a variety of cells including macrophages, fibroblasts, and epithelial and endothelial cells [10, 42] and it is thought to play a key role in several pathophysiological processes, such as atherosclerosis and remodeling after MI. In a large cohort of patients with acute coronary syndrome, an elevated baseline level of CCL2 was associated both with traditional risk factors for atherosclerosis and with an increased risk for death or MI [43]. CCL2 was found to increase in the rat heart during reperfusion but not after 1 or 2 hours of permanent infarction [44]. It was also detected in human cardiac myocytes and human cardiac fibroblasts which revealed CCL2 production under cytokine stimulation, but not under hypoxia, in vitro [45]. In patients with ischemic cardiomyopathy, polymorphism in CCL2 gene (CCL2-2518 A/G single nucleotide polymorphism) was correlated with C-reactive protein (CRP) level [46]. Altogether these findings suggest that this chemokine is more associated with inflammation than with hypoxia/ischemia, being strongly regulated by reperfusion and correlated with a known inflammatory marker such as CRP.

To investigate the mechanism of action of CCL2 and other chemokines in the myocardial response to injury, several preclinical studies were performed in the last two decades. Entman, Frangogiannis, and their coworkers extensively explored this topic and unveiled the connection between CCL2 and cardiac fibrosis in a mouse model of ischemia without necrosis [47]. In particular CCL2, identified in the ischemic myocardium after repetitive cycles of brief ischemia/reperfusion (IR), was associated with increased collagen content, macrophages infiltration, interstitial remodeling, and LV dysfunction, phenomena which were attenuated in CCL2^−/−^ mice. Moreover, fibroblasts isolated from Wild Type (WT) mice exposed to repetitive IR exhibited increased proliferative capacity, which was abrogated in fibroblasts from CCL2 null-hearts. It was found that the profibrotic actions of CCL2 are probably associated with decreased macrophage recruitment since at a molecular level no altered expression of genes associated with cardiac fibrosis was observed. In a different study, using the same experimental model [6], same group also discovered that reactive oxygen species generation in the reperfused myocardium rapidly induced CCL3, CCL4, and CXCL2 upregulation in the venular endothelium in the absence of infarction or irreversible cellular injury. In this study, the chemokines induction correlated with nuclear translocation of NF-*κ*B, c-Fos, and c-Jun in cardiac venules. Since angiogenesis begins in the venular endothelium, these experiments could indicate a potential role of chemokines as angiogenetic agents during a transient ischemia without necrosis. Interestingly, Tarzami and colleagues found a protective effect of CXCL2 dependent CCL2 expression in cardiac ischemia without reperfusion, a survival pathway in target cardiac myocytes themselves. Indeed, CCL2 markedly decreased hypoxia-induced cell death in cultured cardiac myocytes [35]. Among chemokines modulating these processes, CCL2 and its receptor CCR2 seem to be one of the keys of the shift from physiological to pathological conditions in both heart and vessels. CCL2 chemotactically attracts mononuclear cells which are source of fibrogenic mediators like TGF-*β* and Fibroblast Growth Factor. In addition, it induces macrophage synthesis of TGF-*β*1 and collagen. CCL2 is also reported to directly modulate fibroblast activity and phenotype. Moreover, it may be an important mediator in the recruitment of fibroblast progenitors [11].

Activation of the CCL2/CCR2 pathway induces monocyte-mediated inflammation and several related processes; enhanced CCR2 fluorescence intensity on monocytes was observed in hypertensive patients as well as in rat models. In CCR2^−/−^ mice, Angiotensin II- (Ang-II-) induced vascular inflammation and remodeling were blunted compared to WT animals; in a model of fibrosis and hypertrophy secondary to suprarenal aortic constriction, chronic treatment with an antibody against CCL2 led to myocyte hypertrophy without fibrosis and to an improvement in diastolic dysfunction, in the absence of an effect on blood pressure or systolic function. In contrast, control rats showed hypertrophy and reactive fibrosis [48, 49]. Ang-II infusion is a well-established model of myocardial hypertrophy and it has been used to characterize chemokine expression and cellular element involvement. Kuwahara and colleagues showed mRNA-CCL2 upregulation from day 1 to day 3 after treatment, followed by TGF-*β* induction at day 3 to day 28 [49]. The results were confirmed by Sopel and colleagues who measured chemokines expression using quantitative Real Time PCR. They also showed that fibrocytes (fibroblast progenitor cells) were recruited into the myocardium prior to the development of myocardial fibrosis and were probably recruited out of the circulation by gradients of CXCL12, CCL21, and CCL2 [50]. A further demonstration of CCL2 involvement in the progression from hypertrophy to fibrosis originates from a model of genetic deletion of CCL2 in mice [51]. CCL2-Knock-Out (KO) animals receiving infusion of Ang-II were prevented from fibrosis development, induction of collagen, induction of TGF-*β*1, and TNF mRNA expression. In this model, the nonadaptive hypertrophy in the heart clearly required CCL2 induction. In this regard, an interesting clinical study [52] found that patients with heart failure had higher level of circulating CCL2 as compared with healthy controls despite treatment with a high-dose of ACE-inhibitor.

On the other hand, angiogenic and cardioprotective effects of CCL2 have also been reported [35], suggesting distinct effects of this chemokine on remodeling after MI. Morimoto and colleagues investigated the effect of cardiac CCL2 overexpression on LV dysfunction and remodeling in a murine model of MI [53]. The interesting findings were that cardiac overexpression of CCL2 reduced infarct area and scar formation improving LV dysfunction and remodeling, thus suggesting a controversial role of CCL2 in response to MI. At the same time Dewald and colleagues [54] found that inhibition of CCL2 could have the same deleterious effect observed with corticosteroid treatment in patients with MI [55], that is, impairing the repair processes, after injury. Altogether, the role of CCL2 appears therefore to go beyond the simple recruitment/proinflammatory effect and is clearly involved in the fine and critical modulation of cardiac homeostasis after a detrimental insult.

### 2.2. CCL5

Recently, two studies showed the effects of chemokine inhibition on MI. In the first work [7], treatment with a monoclonal antibody anti-CCL5 (RANTES) significantly reduced both infarct size and postinfarction HF in a murine model which was correlated with a decreased leukocyte recruitment within the infarcted hearts. In the second study [9], mice subjected in vivo to left coronary artery permanent ligation and followed up for different times (up to 21 days) showed a beneficial reduction in infarct size as compared to controls when treated with chemokine inhibitors, in particular with CCL5 inhibition, which reduced cardiac injury/inflammation and improved survival. This treatment was associated with a decrease in postinfarction myocardial leukocyte infiltration, reactive oxygen species release, and circulating levels of CXCL1 and CCL2.

### 2.3. CXCL8, CXCL1, and CXCL5

CXCL8 (IL-8) is a chemokine attracting neutrophils, which plays a key role in the early phase of reperfusion injury as well as in heart failure. CXCL8 expression was found to increase in ischemic reperfused myocardium suggesting a role in neutrophil-induced myocardial injury by promoting ligand-specific adhesion to cardiac myocytes [12]. Damås and colleagues showed that circulating levels of CXCL8, CXCL1 (GRO-*α*), and CXCL5 (ENA-78) gradually increased in patients with congestive heart failure (CHF) in parallel with an increase in NYHA functional class [15, 56]. Recently [57], high levels of CXCL8 in patients suffering ST elevation myocardial infarction, complicated by HF, were associated with less improvement in LV function during the first 6 weeks after PCI, suggesting a possible role of CXCL8 in the reperfusion-related injury. In 2001, Chandrasekar and colleagues found that myocardial IR in the rat activates NF-*κ*B and induces neutrophil infiltration via a homologous of CXCL8 in rats called LIX (lipopolysaccharide-induced CXC chemokine) [13]. LIX was found to be expressed by resident myocardial cells during IR and induced in cultured cardiomyocytes by oxidative stress or TNF-*α* via NF-*κ*B activation. Moreover, in a further study, they discovered that LIX, in addition to attracting and activating neutrophils, also amplified the inflammatory cascade, stimulating local production of cytokines that had negative inotropic and proapoptotic effects [58].

 Damås and colleagues [15] examined the circulating levels of CXCL8, CXCL1, and CXCL5 in patients with various degrees of HF, finding that they had a significant increase in the levels of all three chemokines and that both CXCL8 and CXCL1 correlated with the NYHA class.

### 2.4. CXCL12

An additional chemokine involved in cardiac hypertrophy and remodeling is CXCL12, also known as stromal cell-derived factor 1 (SDF-1). This chemokine has a key role in hematopoiesis, cardiogenesis, vasculogenesis, and neurogenesis [59]. The increased expression of CXCL12 in ischemic tissue is considered a beneficial signal attracting stem cells into the heart [17]. Its involvement in cardiovascular homeostasis has been described by several studies.

The main receptor for CXCL12 is CXCR4 which is expressed in several cells including those from megakaryocytic lineage [60]. The main function of CXCL12 interaction with CXCR4 is the mobilization of stem cells from bone marrow.

LaRocca and colleagues [61] demonstrated that CXCR4 physically interacts with the *β*2 adrenergic receptor and modulates the subsequent downstream signaling. CXCL12/CXCR4 are expressed on cardiac myocytes and inhibit contractility in response to the *β*-adrenergic receptor agonist, isoproterenol. Recently [62], it was reported that CXCR4 expression prevented cardiac hypertrophy induced by isoproterenol: CXCR4-KO and WT mice were subjected to chronic administration of isoproterenol for 3 weeks; biochemical as well as echocardiographic and hemodynamic measurements were performed. KO mice showed worsened fractional shortening and ejection fraction as compared with WT animals, as well as upregulation of apoptotic markers and reduced mitochondrial function. There was also an increase in myocardial fibrosis. A CXCR4 gene transfer performed in KO animals reduced apoptosis and improved mitochondrial and cardiac function. However, controversies exist over the protective versus harmful effects of CXCL12 and CXCR4 in models of cardiac injury. Chu et al. [63] found that CXCL12 is constitutively secreted by cardiomyocytes and upregulated by Ang-II and reported an increase in fibroblast migration in a mouse model of dilated cardiomyopathy, suggesting a profibrotic role of this chemokine in the chronically failing heart. In contrast, two authors [64, 65] reported that the CXCL12/CXCR4 axis was the mechanism of the protective effect of apelin-13, an isolated bioactive peptide from bovine extract, and of erythropoietin (EPO), respectively. Apelin-13 increased angiogenesis and cardiac repair by the upregulation of CXCL12/CXCR4 and homing of vascular progenitor cells. EPO, known for its protective properties after IR injury [66], attenuated remodeling, enhanced neovascularization, and diminished apoptotic cells in the peri-infarct area 6 and 30 days after permanent infarction. Moreover, EPO treatment mobilized bone marrow-derived stem cells (BMCs) and enhanced homing of Sca1(+) and CXCR4(+) BMCs toward a CXCL12 gradient into the ischemic myocardium. Interestingly, EPO had no beneficial effects on resident cardiac stem cells. Finally, in an interesting work [67], Hwang and Kloner investigated the potential benefit of simultaneous administration of multiple soluble factors (SFs), including CXCL12*α*, in rats subjected to permanent coronary ligation. The rationale of this work was based on the knowledge that in the clinical setting [68] delivery of a single SF to the ischemic heart had limited effects. Their results revealed no enhancements in cardiac function or reduction in infarct size. Among the possible explanations for the negative results, the authors reported that coronary artery occlusion might have prevented access of intraperitoneal administered SFs to the ischemic region of the myocardium.

### 2.5. CXCL13 and CXCL16

Waehre and colleagues [18] studied the chemokines associated with alteration of the structure in extracellular matrix (ECM), in particular with the function of SLRPs (Small Leucine-Rich Proteoglycans) proteins. SLRPs are proteins capable of binding various types of collagens regulating kinetics, assembly, and spatial organization of fibrils. They found that the interaction between CXCL13 and its receptor CXCR5 was involved in myocardial remodeling, probably regulating proteoglycans and quality of myocardial ECM. This interaction was found to be protective since genetic deletion of the receptor CXCR5 exacerbated dilatation and increased mortality, probably via lack of the increase of SLRPs.

Moreover, in a model of pressure overload dependent right ventricle remodeling, they found [19] that CX3CL1 (also known as fractalkine), CCL5, and CXCL16 regulated expression and posttranslational modifications of SLRPs in cardiac fibroblasts. Therefore these inflammatory mediators may play a role in the development and progression of right-sided myocardial remodeling during pressure overload.

### 2.6. CX3CL1 and XCL1/2

Recently, two chemokines were found to be associated with HF, namely, CX3CL1 and XCL1/2 (also known as Lymphotactin). CX3CL1 is a chemokine with a polypeptide structure different from the typical structure of other chemokines and represents the only member of the CX3C group. It was found to be an independent predictor of mortality in patients suffering HF [69]. Its expression is modulated by TNF-*α* [70] and it is thought to promote cardiac injury and HF by activating MAPKs pathway (mitogen-activated protein kinases pathway) [21]. In a rat heart transplant model, both CX3CL1 and XCL1/2 were found to be involved in the rejection of transplanted hearts secondary to cytomegalovirus infection [22]. The authors suggested that rejection was due to an increase in vascular infiltration of inflammatory cells in the graft through enhanced chemokine expression.

### 2.7. CCL21

CCL21 is a chemokine involved in tissue remodeling and described as “homeostatic” chemokine. Its concentration in serum from HF patients was higher than in controls and was independently associated with mortality in chronic and acute post-MI HF. Interestingly, mice lacking CCL21 receptor, CCR7, showed improved survival and attenuated increase in markers of myocardial dysfunction and wall stress in post-MI HF after 1 week [71] but in the long term presented myocardial dysfunction and increased wall stress. Repair for recovery or decompensated remodeling appears like a crossroad for mediators involved in the process and it is not clear which “input” could tilt the balance in favor of recovery. The autonomic nervous system (ANS) may play a critical role in this setting.

## 3. Crosstalk between Chemokines and Autonomic Nervous System

The best definition for the crosstalk between innate immunity and autonomic nervous system is likely to be* allostasis*, that is, maintaining stability through change [72]. The signals travelling throughout the body create a network of information fine-tuned by the incessant dialog between the systems, aimed at directing the necessary mutual changes. During the late nineties, Armour and colleagues [73] identified intrinsic cardiac ganglia in the heart, especially in the posterior surface of the atria and superior surface of the ventricle, and showed that processes occurring in such an intrinsic cardiac nervous system involve afferent neurons, local circuit neurons, and both sympathetic and parasympathetic efferent postganglionic neurons [74].

When an injury occurs, the heart signals the damage to the nervous system, which responds by leading an orchestrated reaction involving neurohormones, chemokines, cytokines, neuropeptides, and other mediators. After MI, sensory afferent neurons, which respond to hypoxia-induced changes in the heart, are also responsible for pain perception [75] and the sympathetic nervous system modulates activation/proliferation of progenitor cells from bone marrow [76–78].

It is becoming increasingly clear that there is both plasticity and integration at many peripheral anatomical sites in the cardiovascular-immune-neural axis: innate immune system stimulates autonomic nervous system through cytokines, chemokines, platelet-activating factor, and arachidonate metabolites released from immune cells and this activation is integrated at the hypothalamic level [79–82]. Mutually, sympathetic system [83], through the release of norepinephrine (NE), can modulate the functions of immune system cells through receptors expressed on their surface. Other peptides like Neuropeptide Y (NPY) and Substance P (SP) colocalize with NE or are associated with its function and were found to be relevant to heart disease.

SP is a neuropeptide belonging to the family of tachykinins, widely distributed in the nervous system, including the stellate ganglia. It is also localized in the heart and released by sensory nerve endings and immune cells. Macrophages and neutrophils are found to express preprotachykinin gene-I (PPT-I) mRNA and are modulated by SP biological actions.

In the emerging role of chemokines, CXCL2, CCL3, CCL2, and CXCL12 seem to be involved in the crosstalk with the ANS and organs.

In an in vitro model using isolated primary mouse neutrophils, SP primed neutrophils for chemotactic responses to the chemokines CXCL2 and CCL3 and induced both mRNA and protein expression of CCL3 and CXCL2 in neutrophils and upregulated the chemokine receptors CC chemokine receptor 1 (CCR1) and CXC chemokine receptor 2 (CXCR2) [84].

In a recent study it was found [85] that SP seems also to act as a “primer” for cardiac fibroblasts, transiently upregulating genes related to ECM regulation and proteins rather than directly converting fibroblasts to myofibroblasts or increasing collagen synthesis. Moreover, in CD34^+^-derived human mast cells [86], production of CCL2, the potent profibrotic chemokines, was significantly increased by SP treatment, both at protein and at mRNA level. Interestingly vagal nerve stimulation (VNS), which decreases infarct size and adverse LV remodeling and HF in several experimental models, suppressed SP in a rat model of cardiac ischemia [87] and decreased CCL2 and LIX plasma levels in the rat after IR injury [88–90].

CXCL12 seems to be involved in the remodeling of stellate ganglion neurons, a phenomenon occurring in several cardiomyopathies [91–95]. After MI, neural remodeling was associated with an increase in NPY immune-reactivity [96]; NPY, like SP, is a neuropeptide colocalized and coreleased with NE from sympathetic nerve terminals. Wang and colleagues [97] showed that remodeling after MI could be reversed with a therapy combining NPY and mesenchymal stem cells (MSC) in a rat model of MI through a NPY dependent upregulation of CXCL12 gene and others required for mitosis in MSC.

Finally, it was very recently demonstrated [98] that a potent neurotrophin, nerve growth factor (NGF), attracted human bone marrow-derived mononuclear cells similarly to what was observed with CXCL12, used as positive control. In this model NGF promoted progenitor cells homing from the bone marrow to the infarcted heart improving myocardial blood flow and cardiac function.

The interaction between parasympathetic system and innate immunity has been clarified by Tracey at the beginning of this century [99] with the discovery of cholinergic anti-inflammatory pathway, an axis of neuroimmune modulation where acetylcholine (ACh), *α*-7nAChR, and macrophages are the main players with anti-inflammatory properties. When activated, this pathway protected organs, including heart, from the consequences of the ischemic injury [89, 90, 100, 101], decreased levels of CCL2 and LIX after reperfusion injury [88], and downregulated the expression of CCL2, CCL4, CCL5, CCR1, CCR2, and CCR5 in murine autoimmune myocarditis [102].

Both these pathways, immune-sympathetic and immune-parasympathetic, are involved in the beginning and in the progression of heart disease and are reciprocally connected, affecting and modulating each other's functions. It is becoming evident that the mutual crosstalk within the autonomic nervous system between sympathetic and parasympathetic fibers is represented by the ability to reciprocally modulate neurotransmitters release [103] and that these molecules have the ability to modulate the innate immune response.

Despite these intriguing hints, to our knowledge the mutual effects of chemokines, Substance P, NPY, NGF, and ANS in the progression of heart disease remain largely unknown.

It is increasingly clear that the individual autonomic responses already characterized before the development of cardiovascular disease could determine the outcome and the progression of cardiac dysfunction [104–107]. It is conceivable that ANS activation, through neurotransmitters, neurotrophins, and immune mediators, could act as a primary imprinting which orchestrates the response to injury in sense of compensated repair or decompensated progression toward dysfunctional heart disease.

## 4. Inflammation, Chemokines, and Cardiac Arrhythmias

The heart is a highly innervated organ and the link between the autonomic nervous system and ventricular arrhythmias is universally acknowledged: an increased sympathetic tone is a trigger for arrhythmic events in several conditions including patients with ion-channel diseases, ischemic heart disease, and HF. Although the knowledge of inflammation role in the context of cardiac arrhythmia diseases is still scanty, some evidence suggesting its contribution in favoring an arrhythmogenic substrate has been provided.

One of the most intriguing hypotheses is that inflammatory mediators may have a direct effect on the electrical properties of cardiomyocytes. This hypothesis is in agreement with the finding that cytokines and chemokines act as modulators of excitability in neurons [108]. Left cardiac sympathetic denervation (LSCD) is an established additional treatment for patients with long QT syndrome (LQTS) and catecholaminergic polymorphic ventricular tachycardia (CPVT) nonprotected by *β*-blocker (BB) therapy [109, 110]. The presence of inflammatory activity in stellate ganglia of genetically confirmed LQTS and CPVT patients, who underwent LSCD because of malignant ventricular arrhythmias resistant to BB therapy, was evaluated by Rizzo et al. [111]. Inflammatory infiltrates composed of T lymphocytes and macrophages and degeneration of adjacent ganglion cells suggestive of chronic ganglionitis were found in all LQTS/CPVT patients as compared to none of ten control subjects who died in accidents. They proposed that T cell-mediated cytotoxicity toward sympathetic ganglionic cells may increase adrenergic firing and enhance electrical instability in patients already genetically predisposed to ventricular arrhythmias.

A very recent study identified a chemokine that can influence electrical stability and directly modulate action potential duration (AP): CXCL9 has been shown to shorten cardiac AP by reducing sarcolemmal L-type Ca^2+^ current via the G protein-coupled receptor CXCR3, in LV myocytes isolated from male mice [112]. Also in the context of common cardiac diseases such as MI, a proinflammatory condition seems to be associated with an increased risk of developing life-threatening arrhythmias. Elmas and colleagues [14] studied the levels of matrix-degrading metalloproteinases, their inhibitors (TIMPs), and CXCL8, the predominant chemokine interacting with them, in patients with MI complicated or not by ventricular fibrillation (VF). Levels of TIMP-1 and CXCL8 were found to be significantly higher in patients with MI complicated by VF as compared to MI patients without VF. Since high TIMP levels are related to the degree of fibrosis which is a substrate for electrical instability, the presence of these circulating inflammatory mediators may contribute to the occurrence of VF. In addition CXCL8 is a powerful chemoattractant responsible for the recruitment of neutrophils; the adhesion of neutrophils to ischemic cardiomyocytes may result in excitation-contraction disturbances and development of arrhythmias in inflamed cardiac tissue. A high degree of fibrosis is also documented in arrhythmogenic ventricular cardiomyopathy (ARVC), a heart muscle disease characterized by fibrofatty replacement of the right and, less frequently, of the left ventricle. The presence of elevated levels of serum inflammatory mediators including CXCL8, IL-6R, CCL2, and CCL4 and an altered balance between circulating proinflammatory and anti-inflammatory factors have been found in patients with ARVC compared to controls [113]. The same study additionally showed that some inflammatory molecules, either circulating or derived from the myocardium, may lead to disruption of desmosome structure through dislocation of plakoglobin from desmosomes to intracellular space, myocyte injury, and arrhythmogenesis [113].

There is growing evidence of chemokine mediators involvement also in the context of atrial fibrillation (AF). To elucidate the role of circulating inflammatory factors in atrial fibrillation, Wu et al. conducted a meta-analysis based on observational studies [114] and evidenced that the presence of increased inflammation molecules (CRP, IL-6, and TNF-*α*) was significantly associated with a greater AF risk in the general population. High levels of CRP, CCL2, and CXCL8 among patients with AF were found by several investigators [115, 116]. Notably, elevated levels of CXCL8 detected in patients with permanent AF but not in patients with paroxysmal AF suggested a link between low-grade inflammatory reaction and long-lasting AF [116].

The West Birmingham Atrial Fibrillation Project disclosed in a large cohort of AF patients the independent correlation between low plasma levels of CX3CL1 and low risk of major cardiovascular events [117]. Although these studies on AF are only observational and do not explain the molecular mechanisms linking inflammation with AF risk, they suggest the assessment of plasma concentrations of chemokines as a potential application to improve the risk stratification in AF patients.

The presence of inflammatory chemokine mediators appears to be associated with an increased arrhythmic risk both in patients affected by inherited cardiac diseases and in patients with high degree of fibrosis due to nongenetic causes. Inflammatory molecules are significantly more prevalent in patients with heart diseases and documented cardiac arrhythmias than in patients free of arrhythmia. It is likely that in most cases inflammation may act as a trigger in subjects already susceptible to developing arrhythmia.

It must be mentioned that it has not been conclusively shown whether the inflammatory component plays a primary pathogenic role or whether it is rather the consequence of the myocyte injury. The fact that many studies showed ongoing active inflammation in a symptom-free time, remote from any acute event, suggests inflammation may be a trigger of arrhythmia for already susceptible subjects. However, further efforts are required to elucidate the specific molecular mechanisms involving chemokines and other inflammatory mediators in the development of cardiac arrhythmia.

## 5. Chemokines and Dilated Cardiomyopathy

Dilated cardiomyopathy (DCM) is a myocardial disease characterized by an increase in LV size and volume with normal LV wall thickness, associated with a progressive decline of LV contractile function leading to HF [118]. Being the third most common cause of HF worldwide, DCM is the underlying disease leading to up to 25% of CHF cases in the US [119] and the most common cause of heart transplantation. With a prevalence of 1 : 2500, DCM is a common and largely irreversible form of heart muscle disease. Familial cases account for about 20–35% of all DCM and have been reported linked to a diverse group of >20 loci and genes. Although a specific cause for the disease is found in almost 30–35% of DCM cases the others receive an exclusion diagnosis, idiopathic dilated cardiomyopathy (IDC) [120]. Multifactorial genetic traits [121, 122], associated with persistent viral infection and immunological abnormalities including autoimmunity [123], have been postulated as potential causes in the pathogenesis of IDC since the late 1980s. Experimental and clinical data suggest a causal relationship between myocarditis and IDC, confirming that a chronic inflammatory process may underlie the development of IDC [122]. Being the most frequent cardiomyopathy phenotype, DCM appears to be the final common pathway of many cardiac injuries. It appears that the pathways involved with this condition tend to be more disease specific in the initial phases of myocardial injury, but they involve progressively more stereotyped mechanisms in the later phases of myocardial remodeling, progressively leading to DCM and heart failure [124].

Several sources including the natural history of patients with particular conditions such as Chagas disease provided a strong evidence of the process leading from myocardial inflammation to dilated cardiomyopathy [118]. Chagas disease is a well-characterized example of how the immune response meant to avoid the dissemination of* Trypanosoma cruzi*, the parasite responsible for the disease, results in the development of a potentially fatal cardiomyopathy in 30% of the infected cases, years after the acute infection [125]. In the context of this disease,* T. cruzi* triggers an innate immune response involving the production of cytokines (IL-1, IL-6, and TNF-*α*), chemokines (CCL2, CCL5, and CXCL9), and an adaptative Th1 T cell lymphocyte/antibody response. This response leads to control but not to elimination of the infection, causing the progressive development of Chagas cardiomyopathy resulting from this sustained inflammation in susceptible individuals [126]. These susceptible subjects have been identified as patients showing a smaller T reg lymphocyte compartment producing IL-10 and IL-17, resulting in a deficient suppressive activity leading to uncontrolled production of Th1 cytokines and, among others, of the chemokines CCL2, CCL5, and CXCL9 [127, 128]. Several recent studies supported this finding, demonstrating that gene polymorphism associated with increased or decreased production of the above-mentioned chemokines exerts favoring or protective effects, respectively, on the development of Chagas cardiomyopathy [20, 129, 130].

Chemokines causing leukocyte infiltration and cytotoxicity were investigated as potential mechanisms of cardiomyocyte damage also in chronic myocarditis and in sporadic IDC. Macrophages appear to be one of the key cells causing cardiac myocyte damage; CCL2 and other chemokines are a major signal for the recruitment and activation of monocytes/macrophages. In 1995 Seino et al. reported for the first time that the CCL2 mRNA is expressed in the myocardium of patients with IDC [131]. Shortly after, Kolattukudy and colleagues demonstrated in a transgenic murine model that CCL2 leads to increased myocardial leukocyte infiltration and to the phenotype of dilated cardiomyopathy [132]. Therefore, since the late 1990s, CCL2 has been known to be dynamically regulated in DCM and to possibly contribute to the deterioration in LV function [133]. In 2007 the group of Ohe confirmed that the expression of CCL2 was present in all myocardial samples from each of the 13 DCM patients analyzed in their study, but not in those from control subjects. Moreover, the expression level of CCL2 in the myocardium was correlated with the degree of impairment of cardiac function [134]. These experiments proved once more that augmented expression of specific chemokines was associated with adverse ventricular remodeling with their homeostasis with the maintenance of cardiac structure and function. Finally, Göser et al. [135] found that the same chemokines CCL2 and CCL3, acting through their receptors CCR2 and CCR5, are key chemokines for the development of experimental autoimmune myocarditis and proved that inhibition of CCL2 with 7ND gene transfection significantly reduced disease severity in an experimental model of autoimmune myocarditis. However, CCL2 does not exert only unfavorable effects on the dilated failing heart. Interestingly, it may induce also stem cells homing in the dilated myocardium, thus proving that chemokine signaling may be a useful adjuvant for stem cell therapy [136].

Altogether, these experiments show the important role of chemokines, especially of CCL2, in dilated cardiomyopathy and also in myocarditis, providing further clues supporting the existence of a common inflammatory pathway leading to the dilated cardiomyopathy phenotype. Furthermore, some experiments carried out in animal models of autoimmune heart inflammation showed a favorable response in terms of reduction of inflammatory T cells infiltration and cardiomyocyte damage following the administration of some chemokines (e.g., CXCL1, acting through toll-like-receptor 4) [137].

These results show that further knowledge of the mechanisms underlying the chemokine network may be useful to understand the pathophysiological basis of a broad range of conditions leading to DCM and thus to favorably influence the inflammatory conditions underling this widespread heart disease.

## 6. Chemokines in Clinical Practice: A New Tool to Stratify Cardiovascular Risk?

The parameters used to stratify the risk come from clinical practice and from laboratory results. The prediction of the risk of cardiovascular events in apparently healthy people as well as in patients with a previous cardiac event has been the focus of very active research in the last decades. The more the research progresses, the more it becomes clear that the risk stratification process, due to its complexity, can be effectively addressed only through a polyparametric approach. This means that only a combination of a clinical, instrumental, laboratory, and eventually invasive evaluations exploring different biological conditions and pathways can be really helpful in trying to identify and stratify the risk of each individual subject. Although conventional risk prediction algorithms are made available based on the presence of major cardiovascular risk factors identified in diseased populations, authentic and accurate biomarkers of cardiovascular diseases are still lacking. Among them chemokines are of particular interest because as already described they seem to act as a “working-network” involved in all stages of cardiovascular response to injury, in deep linkage with the autonomic nervous system (ANS). Since they work as a network it is difficult to associate only one or few of them with cardiovascular risk. Nevertheless, through the assessment of a predefined setting of chemokines we could have the possibility to evaluate and quantify pathological deviations of cardiovascular system homeostasis even at very initial stages. The incremental prognostic value of a polyparametric approach including an evaluation of inflammatory markers has been clearly demonstrated by Correia and colleagues in 2010 in the setting of acute coronary syndromes [138]. Five cytokines [interleukin- (IL-) 1*β*, IL-6, IL-10, IL-12p70, and tumor necrosis factor- (TNF-) *α* soluble receptor I], five chemokines (CXCL8, CCL5, CXCL9, CCL2, and CXCL10), and CRP were measured at admission in 87 patients with non-ST segment elevation acute coronary syndrome (NSTE-ACS). Individuals who developed events (death, nonfatal acute MI, and refractory unstable angina) during hospitalization had significantly greater levels of CRP, IL-1*β*, IL-12, TNF-*α*, CXCL8, CXCL9, and CCL2 compared with patients without events. These markers were used to create an Inflammatory Score (by inputting one point for each of these variables above the 75th percentile) to be further adjusted for another score, called GRACE. GRACE Score consists of 8 variables, 5 semiquantitative (age, systolic blood pressure, heart rate, plasma creatinine, and Killip class) and 3 dichotomic (positive necrosis markers, ST segment deviation, and cardiac arrest at admission). After adjustment for the GRACE Risk Score (considered as the most accurate one in the setting of NSTE-ACS and largely used in clinical practice), the Inflammatory Score independently predicted events (OR = 1.80; 95% CI = 1.12–1.88). The incorporation of the Inflammatory Score into the GRACE Score promoted a *C*-statistics improvement from 0.77 (95% CI = 0.58–0.96) to 0.85 (95% CI = 0.71–1.0), with a net reclassification improvement of 13% (*p* = 0.007); on the other hand, when only CRP was incorporated into GRACE, the increase on *C*-statistics was not relevant (from 0.77 to 0.80). When we look at the plasmatic levels of each of the 11 markers analyzed in the score, we found very interesting results. CXCL8 was the most varied chemokine between patients with and patients without events; indeed CXCL8 levels were almost 4 times higher in patients with events (median 37 pg/mL versus 10 pg/mL, *p* = 0.003). This is not surprising considering the well-known CXCL8 neutrophils attracting properties and their key role in the development of reperfusion injury. Among the other chemokines analyzed, both CCL2 and CXCL9 levels were significantly higher in patients with events (*p* = 0.02). On the other hand, CCL5 and CXCL10 levels were not different in the two groups. While CXCL10 has never been evaluated in the setting of acute coronary syndromes before, the negative results for CCL5 (lack of association with events) were not expected. However, considering the limited sample size, the possibility of type II error as an explanation of these negative findings could not be ruled out. Finally, the authors clearly demonstrated that although the Inflammatory Score was related with both peak troponin T and peak creatine kinase MB, it remained a significant predictor of events even after the adjustment for these parameters. The results of these studies, although very preliminary, speak in favor of a multimarker strategy (including chemokines) as opposed to a single-marker approach. Unfortunately, the proposed Inflammatory Score (in addition to GRACE Score), although very appealing for the early management of NSTE-ACS, has never been evaluated in other populations and the majority of the studies performed in the last years focused on the evaluation of a single chemokine, therefore missing the central point of the network.

In 2012, De Jager and colleagues studied a larger population of 609 ACS patients (56% infarcted patients with ST elevation or STEMI, 33% non-STEMI, and 11% with unstable angina) and demonstrated a significant prognostic value of a pool of three chemokines (CCL3, CCL5, and CCL18) for the short-term outcome [34]. The average follow-up time was 189 days (189 ± 14.1 days) and patients were monitored for the occurrence of fatal (*n* = 48) and nonfatal (*n* = 22) cardiovascular events (new acute coronary syndrome, ACS, and/or coronary revascularization including both PCI and CABG techniques). High levels of CCL3, CCL5, and CCL18 were found to be independently associated with short-term fatal events in patients with ACS. Furthermore, the risk increased with a growing number of chemokines in the highest concentration tertile, reaching hazard ratio of 2.52 (95% CI: 1.11–5.65) in case of all of them in the upper tertile. Interestingly, no relationships were observed between chemokine levels and the risk of nonfatal events during follow-up and a large difference between the timing of fatal and nonfatal events during follow-up was noticed. Namely, the majority of the fatal events occurred already shortly after the start of follow-up. The median time-to-event of fatal events was merely 5 days, whereas nonfatal events occurred much later during follow-up with a median time-to-event of 120 days. Considering the known predictors of short-term fatal events (including clinical variables and CRP levels), the combination of CCL3, CCL5, and CCL18, if added simultaneously to the model, improved the *C*-statistics from 0.74 to 0.81 (*p* =  0.007). Obviously, the question of whether this increased prognostic value is also of clinical relevance deserves further investigation in studies with larger patient populations.

Altogether, the results of these few studies, although very preliminary, speak in favor of a multimarker strategy as opposed to a single-marker approach. Furthermore, the fact that scores including chemokine were found to be consistently related to acute/subacute clinical outcome rather than to middle term follow-up in the setting of acute coronary syndromes seems to have a strong biological rationale and point out the importance of the shift of the chemokine homeostasis in driving the evolution of an acute myocardial ischemia. Being so, by assessing a pool of chemokine we could have the possibility to predict an adverse biological response (e.g., reperfusion injury) immediately before it is taking place and we could identify high risk patients who may require a more aggressive treatment and/or new experimental interventions (e.g., VNS).

## 7. Conclusion

In the past two decades the evidence suggesting a role for inflammation in the pathogenesis of cardiovascular diseases has been progressively increasing. Most data were related initially to the development of atherosclerosis and to the acute instability of atherosclerotic plaques. As reviewed in this paper, numerous studies now demonstrate a profound involvement of inflammatory processes in a variety of cardiovascular manifestation of diseases such as myocardial ischemia, heart failure, atrial fibrillation, and malignant ventricular arrhythmias. Parallel with the increased understanding of the role of inflammation, data have been gathered on the implication of chemokines in these processes. Chemokines act as a complex network connecting the cardiovascular system with the immune system and the autonomic nervous system. Although the original goal of this extensive crosstalk is to maintain homeostasis and to coordinate the best response to injury, it appears that unbalanced or exaggerated responses may actually lead to a progression of the disease and contribute to an acute and potentially life-threatening manifestation. Many aspects of the involvement of chemokines must still be elucidated before their role can be considered clarified. Moreover, further data are required before their evaluation can be used clinically in the prediction of the disease, in risk stratification of patients, and possibly in paving the way to new therapeutic strategies. In many circumstances the “chicken and egg” question has not been solved, being still uncertain whether specific chemokines are involved in favoring the disease or rather are the result of the disease, being triggered by it. Also, it has become increasingly evident that chemokines represent a very complex network and that it would be naïve to believe that by simply assessing a single one of them a significant improvement in the comprehension of a disease process or in the risk stratification of individual subjects may be derived. This goal may be possibly achieved using a multidimensional evaluation in which multiple markers including panels of appropriately selected chemokines are assessed together. Should we achieve this goal we would have probably reached the* heart* of the problem for a better understanding, diagnosis, and therapeutic strategy of cardiovascular diseases.

## Figures and Tables

**Table 1:** List of chemokines relevant in cardiovascular diseases.

Chemokine (systematic name)	Colloquial name	Receptor	Main function	Associated cardiac disease
*CC chemokines*				
CCL2	MCP-1	CCR2	Recruitment of monocytes, memory T cells, dendritic cells	Cardiac ischemia, reperfusion injury, remodeling, fibrosis heart failure [10]
CCL3	MIP-1*α*	CCR1	Neutrophils activation, induction of cytokines synthesis	Cardiac Ischemia [6]
CCL4	MIP-1*β*	CCR1, CCR5	Neutrophils activation, induction of cytokines synthesis	Cardiac ischemia [6]
CCL5	RANTES	CCR5	Leukocyte recruitment	Cardiac ischemia [7]
CCL11	Eotaxin	CCR2-3-5	Modulation of macrophage function in the plaque	Atherosclerosis cardiac ischemia [8]
CCL18	PARC	Under investigation (GPR30, CCR8, PITPNM3)	Functions primarily involved with recruitment of the adaptive immune system	Acute coronary syndromes [34]
CCL21	—	CCR7	Vascular inflammation, cell proliferation, matrix remodeling	Heart failure [16]

*CXC chemokines*				
CXCL1	Gro-a, GRO1, NAP-3, KC	CXCR2	Neutrophil chemoattractant activity	Cardiac postinfarction [9]
CXCL2	MIP-2	CXCR2	Chemotactic for neutrophils and hematopoietic stem cells	Cardiac reperfusion injury [6], cardiac infarction [35]
CXCL4	PF4 (Platelet Factor 4)	CXCR3	Chemotactic for neutrophils, fibroblasts, monocytes	Atherosclerosis [5]
CXCL5	ENA-78	CXCR2	Chemotaxis of neutrophils, angiogenic properties	Heart failure [15]
CXCL8	IL-8, NAP-1, MDNCF, GCP-1	CXCR1, CXCR2	Chemotactic for neutrophils	Cardiac ischemia, reperfusion injury [12], cardiac arrhythmias [14], heart failure [15]
CXCL9	MIG	CXCR3	T cell chemoattractant	Dilated cardiomyopathy [20]
CXCL10	IP-10, CRG-2	CXCR3	Monocyte/macrophages, chemoattractant, T cell adhesion to endothelial cells, angiogenesis	Pressure overload, remodeling [19] Dilated cardiomyopathy [20]
CXCL12	SDF-1	CXCR4	Mobilization of stem cells from bone marrow	Hypertrophic cardiomyopathy, fibrosis and remodeling, HF [17]
CXCL13		CXCR5	Homing of B cell, monocyte activation apoptosis	Matrix remodeling [18]
CXCL16	—	CXCR6	Migration of T and NKT cells, stimulating production of SLRPs from fibroblasts	Matrix remodeling and heart failure [19]

*CX3C chemokine*				
CX3CL1	Fractalkine, neurotactin, ABCD-3	CX3CR1	T cell and monocytes chemoattractant	Heart failure rejection of a transplanted heart [21, 22]

*C chemokine*				
*XCL1 and 2*	Lymphotactin a, SCM-1a, ATAC and Lymphotactin *β*, SCM-1*β*	*XCR1*	T cell chemoattractant	Heart transplantation [22]

## Abbreviations

AF: Atrial fibrillation
ANS: Autonomic nervous system
CPVT: Catecholaminergic polymorphic ventricular tachycardia
CRP: C-reactive protein
CVD: Cardiovascular disease
CABG: Intervention-coronary artery bypass grafting
DCM: Dilated cardiomyopathy
HF: Heart failure
IDC: Idiopathic dilated cardiomyopathy
IR: Ischemia and reperfusion
LQTS: Long QT syndrome
LV: Left ventricle
NYHA: New York Heart Association
NE: Norepinephrine
NGF: Nerve growth factor
NPY: Neuropeptide Y
NSTE-ACS: Non-ST segment elevation acute coronary syndrome
PCI: Percutaneous coronary intervention
ROS: Radical oxygen species
SP: Substance P
VNS: Vagal nerve stimulation
*α*-7nAChR: Alpha-7-n acetylcholine receptor
VF: Ventricular fibrillation.
