# Corrigendum to “Chemokines and Heart Disease: A Network Connecting Cardiovascular Biology to Immune and Autonomic Nervous Systems”

**DOI:** 10.1155/2018/4128049

**Published:** 2018-01-10

**Authors:** Veronica Dusi, Alice Ghidoni, Alice Ravera, Gaetano M. De Ferrari, Laura Calvillo

**Affiliations:** ^1^Department of Cardiology and Cardiovascular Clinical Research Center, Fondazione IRCCS Policlinico San Matteo, 27100 Pavia, Italy; ^2^Center for Cardiac Arrhythmias of Genetic Origin, IRCCS Istituto Auxologico Italiano, 20095 Milan, Italy; ^3^Department of Molecular Medicine, University of Pavia, 27100 Pavia, Italy; ^4^Institute of Cardiology, Department of Medical and Surgical Specialties, Radiological Sciences, and Public Health, University and Civil Hospital of Brescia, 25123 Brescia, Italy

In the article titled “Chemokines and Heart Disease: A Network Connecting Cardiovascular Biology to Immune and Autonomic Nervous Systems” [1], there was an error in Figure 2. The corrected figure is shown below.

## Figures and Tables

**Figure 1:**
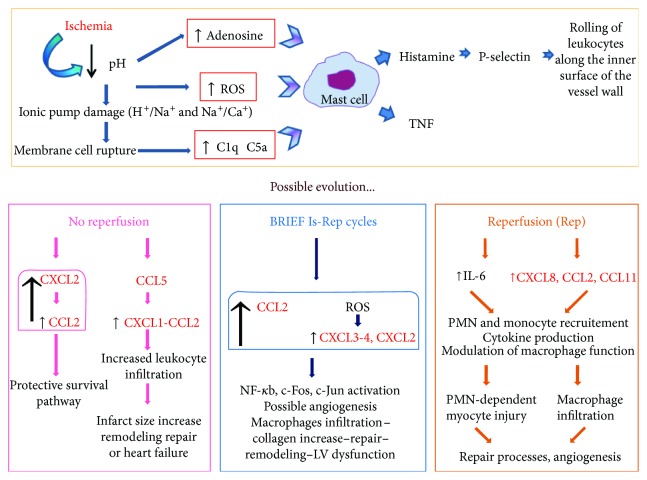
Metabolic changes triggered by ischemic insult and possible evolutions of the injury with the crosstalk between chemokines. The pH decrease, provoked by ischemia, is the event turning on the process. The cell membrane is damaged and debris activates the classic complement pathway in the infarcted myocardium. ROS, adenosine, and complement activate mast cells to produce TNF and histamine, leading to leukocyte recruitment from the vessels. Depending on the presence or the absence of reperfusion, there is a different crosstalk between chemokines aimed at restoring the balance. Dysregulated or exaggerated responses may actually lead to a progression of the disease (see text for details, chemokines in red).

## References

[1] Dusi V., Ghidoni A., Ravera A., De Ferrari G. M., Calvillo L. (2016). Chemokines and heart disease: a network connecting cardiovascular biology to immune and autonomic nervous systems. *Mediators of Inflammation*.

